# Perspectives on Molecular Biomarkers of Oxidative Stress and Antioxidant Strategies in Traumatic Brain Injury

**DOI:** 10.1155/2014/723060

**Published:** 2014-02-13

**Authors:** André Mendes Arent, Luiz Felipe de Souza, Roger Walz, Alcir Luiz Dafre

**Affiliations:** ^1^Department of Biochemistry, Federal University of Santa Catarina, Biological Sciences Centre, 88040-900 Florianópolis, SC, Brazil; ^2^Faculty of Medicine, University of South Santa Catarina (Unisul), 88137-270 Palhoça, SC, Brazil; ^3^Neurosurgery Service, São José Regional Hospital (HRSJ-HMG), 88103-901 São José, SC, Brazil; ^4^Applied Neurosciences Centre (CeNAp) and Department of Medical Clinics, University Hospital, Federal University of Santa Catarina, 88040-900 Florianópolis, SC, Brazil

## Abstract

Traumatic brain injury (TBI) is frequently associated with abnormal blood-brain barrier function, resulting in the release of factors that can be used as molecular biomarkers of TBI, among them GFAP, UCH-L1, S100B, and NSE. Although many experimental studies have been conducted, clinical consolidation of these biomarkers is still needed to increase the predictive power and reduce the poor outcome of TBI. Interestingly, several of these TBI biomarkers are oxidatively modified to carbonyl groups, indicating that markers of oxidative stress could be of predictive value for the selection of therapeutic strategies. Some drugs such as corticosteroids and progesterone have already been investigated in TBI neuroprotection but failed to demonstrate clinical applicability in advanced phases of the studies. Dietary antioxidants, such as curcumin, resveratrol, and sulforaphane, have been shown to attenuate TBI-induced damage in preclinical studies. These dietary antioxidants can increase antioxidant defenses via transcriptional activation of NRF2 and are also known as carbonyl scavengers, two potential mechanisms for neuroprotection. This paper reviews the relevance of redox biology in TBI, highlighting perspectives for future studies.

## 1. Introduction

According to the World Health Organization, traumatic brain injury (TBI) is the leading cause of death in young adults. TBI will surpass many diseases and will become the third cause of death and disability in the general population by the year 2020 [1, 2]. The high medical costs of these patients can compromise the entire health care system [3].

The International Mission for Prognosis and Analysis of Critical Trials in TBI (IMPACT study) has developed a prognosis calculator based on admission data of more than 8500 patients [4]; validation studies has shown it to perform with reasonable accuracy [5]. However, the predictive power of this outcome calculator can be improved by the use of brain injury biomarkers [6], while post-TBI prognosis itself can be improved through the development of new neuroprotective strategies. To determine a good biomarker it seems essential that pathophysiologic mechanisms involved in the initial phase of TBI should be known in detail, while a more extended understanding of regulatory mechanisms is also required for effectively promoting neuroprotection.

## 2. Pathophysiology

### 2.1. Clinical Parameters

The clinical outcomes of TBI are directly related to the severity of the primary and secondary lesions sustained by the patient. Primary lesions are those related to the initial impact (lacerations, contusion, fractures, and diffuse axonal injury). Secondary lesions are those which developed after the initial trauma, including hematomas, edema, and pathological processes cascades that cause ischemia resulting in a worsening of the clinical condition [7]. The development of the secondary injury in TBI is a complex process involving oxidative stress, glutamate excitotoxicity, inflammatory damage, and the toxicity of metabolites that can be disseminated by the circulatory system [8, 9].

The therapeutic management of intracranial trauma aims to avoid the development of secondary lesions, and to this end, the control of physiological parameters such as cerebral perfusion pressure (CPP), intracranial pressure (ICP), and cerebral blood flow (CBF) is crucial to minimize ischemia and tissue damage [10]. The clinical use of CPP as a clinical parameter is based on theoretical suggestions indicating that optimal cerebral blood flow is necessary to meet the metabolic needs of the injured brain [11]. The therapeutic management goal is to keep the CBF stable and maintain a balance between CPP and ICP in order to rescue the ischemic penumbra area. Cells in this area are potentially salvable; therefore, they comprise the most essential area for medical intervention, making the prevention of secondary insults in this region crucial for a better outcome [12].

### 2.2. Brain Swelling

Among secondary injuries, cerebral edema is of special significance, as it can greatly aggravate brain damage and is the main condition related to increased ICP, excluding conditions potentially leading to surgical interventions, such as hematoma and contusion. Increased ICP leads to a decrease in CPP and CBF, worsening tissue damage as a consequence of brain ischemia. Edema occurs by two basic mechanisms, cytotoxic edema related to the depletion of cell energy and vasogenic edema related to disruption of the brain-blood barrier (BBB) [13, 14].

In vasogenic brain edema, BBB integrity is compromised by mechanical or autodigestive disruption, or functional breakdown of the endothelial cell layer of brain vessels, which is critical for maintenance of the BBB. Disintegration of the cerebral vascular endothelial wall allows for uncontrolled ion and protein transfer from the intravascular to the extracellular (interstitial) brain compartment, leading to fluid accumulation, which increases the volume of the extracellular space [15, 16]. The intact BBB prevents diffusion of most water-soluble molecules above 500 Da [17]. However, when the BBB is disrupted, brain-related proteins can be measured in the peripheral circulation [18, 19]. The BBB leakage associated with TBI not only allows brain-related molecules to reach the bloodstream but also permits molecules from the periphery to enter cerebrospinal fluid (CSF). Both situations, either peripheral proteins entering the CSF or CSF leakage of proteins, can be used as biomarkers of TBI [20, 21].

Cytotoxic brain edema is characterized by intracellular water accumulation inside neurons, astrocytes, and microglia. This pathology is caused by an increased cell membrane permeability, ionic pump failure due to energy depletion, and intracellular accumulation of osmotically active solutes [16, 22]. This edema-driven energy impairment generates an “ischemia-like” pattern, which increases glycolysis flux, leading to lactic acid accumulation, associated with increased membrane permeability, intensifying edema, and the establishment of a destructive positive feed-back loop.

The next step of this pathophysiological cascade is characterized by excitoxicity. TBI is associated with a massive release of excitatory amino acid neurotransmitters, particularly glutamate [23, 24]. The extracellular glutamate availability affects neurons and astrocytes and results in overstimulation of ionotropic and metabotropic glutamate receptors, increasing Ca^2+^, Na^+^, and K^+^ influxes [25, 26]. Although these events trigger catabolic processes, the cellular attempt to compensate for ionic gradients increases Na^+^/K^+^-ATPase activity and therefore metabolic demand, creating a vicious circle of flow-metabolism uncoupling. This condition can destroy vascular and cellular structures and, ultimately, induces necrotic or programmed cell death [27].

## 3. Reactive Oxygen Species and Traumatic Brain Injury

Reactive oxygen species (ROS) and reactive nitrogen species (RNS) are generated during normal physiological processes. They are highly reactive molecules which can cause damage to key cellular components such as DNA, lipids, and proteins. Under physiological conditions, the endogenous defense system is able to prevent the formation of or scavenge these harmful molecules, protecting tissues from oxidative damage. In TBI there is a considerable increase in the production of free radicals, supporting the idea that oxidative stress plays a decisive role in the pathology [28, 29].

It is generally believed that 1-2% of the oxygen reduced by mitochondria is converted to superoxide anion (O_2_^•−^) at the level of complex I or at the level of ubiquinone [30–32]. However, certain enzymatic components are loosely associated with the inner mitochondrial membrane and, under conditions of cellular stress, can be released or inactivated, greatly diminishing the reducing capacity of the electron transport chain (ETC). The electrons will subsequently be monoelectronically donated to oxygen (O_2_), yielding increased production of O_2_^•−^. Another important source of mitochondrial O_2_^•−^depends on Ca^2+^ influx, often secondary to glutamatergic excitotoxicity, which leads to structural alterations of the inner mitochondrial membrane. These alterations may increase ROS formation due to disorganization of the ETC [33]. Under severe Ca^2+^ loads, however, opening of the mitochondrial permeability transition pore (mPTP) results in the extrusion of mitochondrial Ca^2+^ and other high- and low-molecular weight components. This catastrophic event discharges and uncouples the ETC, preventing ATP production, which can lead to necrotic or apoptotic cell death [34].

Other sources of free radicals in TBI, in addition to mitochondrial dysfunction and excitotoxicity mediated by glutamate, include the formation of bradykinin. This cytokine can activate phospholipase A2, releasing arachidonic acid that can serve as a source of free radicals [35, 36]. Arachidonic acid may also facilitate NADPH oxidase activity, thus further increasing ROS production [37]. Apart from increasing arachidonic acid production from membrane phospholipids, bradykinin can induce free radical production by causing a Ca^2+^ overload [38]. Another source of ROS in TBI may be macrophages/microglia and neutrophils activated as part of an inflammatory process triggered by the initial injury [28].

The extremely short half-life of ROS in biological systems makes direct measurement virtually impossible in a clinical setup. Therefore, several indirect approaches have been used for the estimation of ROS. These include measurement of (1) products of enzymes known to coproduce ROS, (2) stable adducts formed by the reaction between ROS and endogenous or exogenous trapping agents, and (3) endogenous scavengers [39]. The brain is highly sensitive to oxidative stress because this 1300 g organ consumes about 20–30% of inspired oxygen and contains high levels of both polyunsaturated fatty acids and redox transition metals, making it an ideal target for a free radical attack [40].

Free radical acting on polyunsaturated fatty acids leads to the formation of highly reactive electrophilic aldehydes, including malondialdehyde (MDA), 4-hydroxy-2-nonenal (4-HNE), which are the most abundant products, and acrolein, the most reactive product [41–43]. Lipid peroxidation products, such as 4-HNE and thiobarbituric acid-reactive substances, are studied in order to identify an oxidative stress condition in experimental models of TBI [44–46]. Reactive aldehydes are a noxious byproduct of lipid peroxidation, which, among other things, increase BBB permeability [47], contribute to cytoskeletal changes in neurons [48], and affect glucose transport across membranes [49, 50].

Oxidative stress also damages nucleic acids, both by inducing DNA fragmentation, which consists of single- and double-stranded DNA breaks, the latter being irreversible and occurring a few hours after brain injury [51, 52], and via oxidative damage leading to modification and loss of DNA bases. The predominant base modification used as an index of DNA-oxidative damage is 8-hydroxy-2′-deoxyguanosine (8-OHdG). Single-strand breaks and base oxidation can be repaired [53], but an inefficient DNA repair may delay neurobehavioral recovery after TBI [54]. Some experimental work has demonstrated that oxidative damage to DNA occurs early in TBI and can be targeted by therapeutic strategies [55, 56]. However, intriguingly, in a study using administration of the ROS scavenger alpha-phenyl-N-tert-butyl-nitrone, the authors found an improvement in memory (water maze task) accompanied by a paradoxical increase in neuronal DNA fragmentation. These data suggest that DNA fragmentation would not be a good marker for TBI [57]. On the other hand, immunohistochemical analysis of DNA damage markers in autopsy samples has suggested the validity of single-strand breaks as markers of TBI. Given these findings, DNA single-stranded breaks may be more helpful when used in conjunction with other biomarkers such as glial fibrillary acidic protein (GFAP) and basic fibroblast growth factor (bFGF) in providing clues on different cell death mechanisms of succeeding TBI [58]. However, there is a need for guidelines to support the use of DNA modifications as a marker of TBI.

## 4. Antioxidant Defenses

Antioxidants act in a concerted fashion in the normal brain and can be classified into two major groups: enzymes and low-molecular-weight antioxidants. The enzymes include a number of proteins, including SOD, catalase, and peroxidases, as well as some supporting enzymes. The expression of these enzymes and their activity diverge in different brain regions [59]. The protective role of endogenous antioxidant enzymes in ischemic brain injury has been well established in the literature [60]. Trauma not only interferes with the regulation of antioxidant mechanisms but may also convert these mechanisms into prooxidative ones through its ability to disrupt cell compartmentalization [61].

The low-molecular-weight antioxidants comprise a concerted system of water- and lipid-soluble molecules like glutathione (GSH), ascorbic acid, histidine-related compounds (carnosine, homocarnosine, and anserine), melatonin, uric acid, lipoic acid, and tocopherols [59]. These are extremely important in minimizing oxidative stress. However, cells can synthesize only a limited number of these molecules (e.g., GSH and carnosine). The majority of low-molecular-weight antioxidants are derived from dietary sources. The concentration of ascorbate, which has a relatively high antioxidant potential, is unusually high in the brain, [62]. However, there is a very limited literature about the importance of this antioxidant in TBI. Brain ascorbic acid was shown to be decreased in experimental blast-induced TBI and has been associated with decreases in GSH and protein thiols and an increase in oxidative markers [63–65].

Cells are equipped with enzymes that can eliminate peroxidation end products, such as the aldehyde 4-HNE. These enzymes include aldehyde dehydrogenases, aldo-keto reductases, carbonyl reductase, and glutathione S-transferases (GST) [66]. GST is an enzyme that displays glutathione peroxidase activity and has, by far, the highest detoxifying capability of highly toxic aldehydes such as 4-HNE. Naturally occurring variation in the GST expression affects neurodegeneration after experimental TBI, confirming the importance of lipid peroxidation as an important pathophysiological mechanism in TBI [67].

## 5. Biomarkers of TBI

Biochemical biomarkers can be analyzed from serum or whole blood. Disadvantages of this approach include lack of specificity to brain tissue, high variability in the extent of BBB disruption, and low sensitivity to early injury. Alternatively, we can assess CSF markers that may be more specific to central nervous system (CNS) tissue and sensitive to early injury, although CSF collection is more invasive and not routinely available in medical practice. Another option would be the measurement of parenchymal interstitial fluid via microdialysis. High lactate to pyruvate ratio, increased levels of glycerol, and low levels of glucose have been correlated with poor clinical outcome. However, cutoff points for certain parameters are broad and poorly validated. One rational approach that may lead to identification of blood or CSF markers would be to evaluate biochemical processes known to play a central role in CNS injury, including markers of inflammation, glial activation, neuronal dysfunction, and oxidative stress [68].

TBI biomarkers can reveal structural brain damage but are also markers of secondary injury cascades. TBI promotes genomic, proteomic, and lipidomic changes, as well as oxidative stress, neurotransmitter dysfunction, mitochondrial failure, and other processes [69]. Therefore, TBI biomarkers can also indicate appropriate therapeutic strategies to minimize secondary brain injury and long-term sequelae. Using TBI biomarkers increases the predictive value of outcome calculators and improves the development of individualized treatment courses, thereby reducing outcome severity [70]. The detection of oxidatively modified biomolecules could be used as biomarkers to demonstrate the extension of cellular damage or changes in the cascade of secondary brain damage and repair [39, 65].

Several molecules have been investigated as biomarkers of TBI. CNS-specific molecules include creatine kinase [71, 72], lactate dehydrogenase [71, 72], glial fibrillary acid protein (GFAP) [72–75], myelin basic protein [72, 76], neuron-specific enolase [77–79], S-100*β* protein [72, 75, 80], brain and heart type fatty acid-binding proteins [81], tau proteins [82, 83], brain-derived neurotrophic factor (BDNF) [84, 85], and ubiquitin carboxy-terminal hydrolase-L1 [86, 87]. The most commonly used inflammatory serum biochemical markers include heat shock protein 70 kDa (Hsp70) [88], regulated on activation normal T cell expressed and secreted (RANTES) [89], tumor necrosis factor alpha (TNF-*α*), and interleukins [90].

*N*-acetylaspartate (NAA), a nervous system-specific metabolite, is synthesized from aspartate and acetyl-coenzyme A in neurons. NAA has been shown to be a marker with diagnostic relevance in monitoring metabolic state after TBI [65]. NAA is the second most concentrated metabolite in the brain after the amino acid glutamate. It is only detected in adult brain neurons and is synthesized in the mitochondria. NAA and ATP metabolism appear to be linked indirectly, whereby acetylation of aspartate may facilitate its removal from neuronal mitochondria, thus favoring conversion of glutamate to *α*-ketoglutarate, which can enter the tricarboxylic acid cycle for energy production [91]. Accumulating evidence in the last decade suggests that NAA is a marker of mitochondrial dysfunction in the brain. A close relationship has been demonstrated between trauma severity, depression of energy metabolism, and NAA [92, 93]. Alterations in NAA levels have also been demonstrated in many cerebral pathologies, and their noninvasive *in vivo* quantification by ^1^H-NMR spectroscopy makes them a particularly attractive biomarker [94–96].

The activity of the most studied antioxidant enzymes in TBI, CAT, SOD, and GPx presents random changes in animal models of TBI. In some cases an increase [97, 98] and in others a decrease [99, 100] or no change [101, 102]. The heterogeneity in sampling time points and animal models may be related to this lack of consistency across studies. Whatever the reason, to date there is little consistency in data regarding antioxidant enzymes to justify their use as markers of TBI and as a predictive tool of outcome.

Glutathione, the major nonprotein thiol of cells, usually decreases after TBI in rats [64, 99] but not in mice [64, 99, 103–105], demonstrating species-specific characteristics that should be taken into account when using information obtained from animal studies.

The peroxidation of membrane lipids can change the membrane function by modifying its fluidity, permeability, metabolic processes, and ionic equilibrium [106]. Damage to mitochondrial membranes can also increase the production of ROS, besides generating mitochondrial dysfunction. The damage to brain membrane lipids is an early event. In thirty minutes after trauma, higher levels of MDA and 4-HNE can be detected, whose levels are maintained elevated 72 h after the injury onset [107–109]. Most studies analyzing the oxidative damage to lipids in animal models find a correlation between this parameter in conjunction with cognitive damage, installation of edema, and volume of injury. These data suggest that the damage to lipids of biological membranes can be an important event in this pathology [110, 111].

Oxidative damage to DNA is also an early event in TBI. In animal models, the appearance of 8-OHdG, oxidative marker of DNA damage, is increased within the first 15 minutes after trauma, demonstrating that the TBI-induced ROS production can interfere with the integrity of DNA [55]. On the other hand, the administration of edaravone, an antioxidant, proved to be capable of blocking the DNA damage, resulting in improvement in behavioral tests in mice [112–114].

It has been shown that after a mild TBI, rats showed increased protein carbonylation, another marker of oxidative stress. This event correlated with poor performance in behavioral tests (Morris water maze test), accompanied by decrease in the levels of the neurotrophic factor BDNF. These alterations were neutralized by the administration of antioxidant vitamin E, showing that oxidative damage to proteins may have a key role in neuronal death in TBI [115]. Evidence also points to the involvement of peroxynitrite on the pathophysiology of TBI. A number of studies showed precocious (1 h) increase in protein nitration markers, such as 3-nitrotyrosine [116, 117]. By contrast, protein carbonyl and lipid peroxidation levels were increased in mild TBI in different parts of the brain. However, these oxidative changes were not observed, or even decreased, in severe TBI [46]. These results were somewhat corroborated by Schwarzbold and collaborators, showing that the oxidative damage does not completely correlate with the degree of trauma severity [98].

Several lines of evidence point to the occurrence of oxidative stress in brain trauma. Bayir and collaborators showed that children, who have suffered severe TBI, presented a clinical evolution that is marked by progressive impairment in antioxidant defenses and increase in lipid peroxidation. These findings correlate with the clinical evolution of these patients. Among the endpoints analyzed authors relate ascorbate depletion in the cerebrospinal fluid, followed by the ascorbyl radical formation, in addition to a decrease in the levels of GSH and antioxidant capacity [118]. Arachidonic acid derivatives used as markers of lipid peroxidation, such as F2-isoprostanes, are found at high levels in cerebrospinal fluid after TBI. In fact, they are positively correlated with neuron-specific enolase, a marker of neuronal damage [119, 120]. However, the clinical applicability of this technique is limited, and not all patients can be analyzed due to the need of sampling of CSF.

The 8-iso-prostaglandin F2*α* (8-iso-PGF2*α*) is derived from either enzymatic, by cyclooxygenase, or nonenzymatic oxidation of arachidonic acid [121]. This isoprostane is considered an excellent marker of oxidative stress *in vivo*. Recent data correlate plasma levels of this marker with the Glasgow coma scale (GCS). This finding is very important because it would be a predictive factor of mortality and outcome with sensitivity similar to GCS [122, 123]. Thus, this marker can be a tool in predicting the prognosis of patients with TBI and used as a marker of lipid peroxidation which can be dosed in peripheral blood sample.

Nitrosative stress, mainly detected by the presence of 3-NT, has been demonstrated in TBI [124, 125]. Darwish and collaborators [126] detected 3-NT in the CSF of 7 out of 10 TBI patients, but it was not found in control samples. High levels of 3-NT were also associated with a negative neurological outcome measured by the Glasgow outcome scale, but again, a marker obtained from the cerebrospinal fluid has limited applicability.

In spite of solid evidence of increased oxidative stress markers in TBI, the correlation of these changes with the severity of TBI, as measured by the GCS, is still controversial. For example, higher levels of lipid peroxidation, decrease in the levels of GSH, and increased activity of SOD were observed in erythrocyte of patients with severe TBI, compared to patients who are victims of mild TBI [127–129]. Another work of the same group, however, showed increased lipid peroxidation in erythrocytes of patients with TBI, but this was not correlated with GCS [130]. Plasma levels of lipid peroxidation and protein carbonylation were also not predictive factors associated with hospital mortality or as cognitive impairment in TBI patients [131, 132].

Although the literature demonstrates the unequivocal correlation of markers of oxidative stress with trauma, the correlation of levels of 4-HNE and MDA with the outcome of the TBI presents divergent data necessitating further studies to determine if there is, in fact, some association with the prognosis and outcome. This divergence may be related to a higher incidence of ischemia reperfusion in the mild trauma compared to severe trauma [46]. One of the main sources of ROS in TBI occurs during tissue reperfusion after ischemia, which is an event secondary to trauma. In severe trauma, however, there is a large area of primary tissue injury causing extensive cell death. However, in mild trauma more viable cells that can benefit from reperfusion present higher ROS production, which may favor the use of markers of lipid peroxidation as predictive biomarkers of outcome.

Overall, it seems that the oxidative stress occurs simultaneously in various conditions during and after the brain trauma, but its correlation with the severity and outcome is still not very well understood. Despite all the lines of evidence that indicate a central role of ROS in the cascades of secondary damage, the actual implication of them for neuronal death is not yet clear. As a general picture from the literature data, oxidative damage is not directly correlated with the severity of TBI. An area of study that may shed light on the use of oxidative stress biomarkers needs to take into account changes in redox signaling pathways, besides assessing direct oxidative damage to macromolecules, which may regulate determining factors that contribute to the final outcome.

## 6. Oxidative Stress-Mediated Factors in TBI

Age is another important factor in TBI. Elderly patients with TBI have a worse clinical outcome when compared to younger patients [133]. The influence of oxidative stress on the development of aging has also been demonstrated. For instance, older rats showed higher levels of lipid peroxidation end product (4-HNE), along with lower antioxidant defenses [134]. A worse cognitive outcome, also demonstrated in older rats, was correlated with lower mitochondrial antioxidant enzymes and high levels of 8-OHdG, 4-HNE, single-strand DNA breaks, and malondialdehyde, suggesting the involvement of ROS [135].

Post-TBI complications such as seizures and cognitive deficits also appear to be mediated by ROS in experimental models [136–138]. There is evidence that suggests that antioxidant therapy may reduce lesions induced by oxidative free radicals in some animal seizure models [139].

Interestingly, physical conditioning seems to decrease oxidative damage to lipids formed after TBI in rats [140]. Both, the use of amphetamines, as well as physical exercise can reduce oxidative damage after TBI [141]. There is preclinical evidence suggesting that exercise can improve cognitive outcomes in experimental TBI [142].

## 7. Antioxidant Strategies for Neuroprotection

The relationship between oxidative stress and TBI has generated considerable interest in the development of antioxidant therapies for neuroprotection. Despite the promising results in the treatment of TBI in animal models, evidence of successful antioxidant therapy in clinical practice is limited [143]. There are a number of drawbacks to the use of exogenous antioxidants, for example, the limited penetrability through the BBB, the rapid metabolism and instability of these compounds, short therapeutic windows, and a very narrow therapeutic dosage range, resulting in toxicity at higher doses [143, 144]. Despite these limitations, natural antioxidants and modified antioxidants are promising candidates for future drugs to treat TBI.

### 7.1. Free Radicals Scavengers

Modified SOD has been used in different models as a scavenger of O_2_^•−^, as this radical has increased production after TBI. Lecithinized SOD displays high affinity to cell membranes and was able to increase neuronal counts in an animal model of TBI [145, 146]. Another superoxide scavenger, OPC-14117, reduced edema formation, neurological deficit, and infarct area caused by TBI [147–149].

Polyethylene glycol-conjugated superoxide dismutase (PEG-SOD) has been tested in clinical trials. Initially, some benefits in a phase II study of PEG-SOD were demonstrated, including the reduction of persistent vegetative state and death outcomes when compared to placebo [150], but these observations were not confirmed in the phase III trial. Failures such as these have been attributed to a narrow therapeutic window [151]. In this way, strategies aimed at directly scavenging ROS are restricted due to the extremely short half-life of free radicals and the small therapeutic window in a TBI event.

Nitrones and derivatives are used in neuroprotection models. Tempol (4-hydroxy-2,2,6,6-tetramethylpiperidin-1-oxyl) is a long-known radical scavenger that displays neuroprotective properties [152], including maintaining mitochondrial function in brain cells affected by TBI [153]. Other radical scavengers, such as *α*-phenyl-N-*tert*-butyl nitrone (PBN), or its sulfated form, are able to improve cerebral blood flow and glucose metabolism, which are accompanied by improvement in neurologic scores [154–157]. However, the therapeutic window is relatively short due to initial burst of ROS in the first moments after the trauma. Therefore, strategies to increase the therapeutic windows are necessary to allow the efficient use of these radical scavengers [158].

The antioxidant *α*-tocopherol is hydrophobic and is able to prevent membrane lipids from undergoing peroxidation. The use of *α*-tocopherol contributed to a lower lipid peroxidation [159], edema [160, 161], and improvement in histological markers in experimental models of TBI [162].

Melatonin displays radical scavenger properties toward ^•^OH, O_2_^•−^, singlet oxygen, and peroxynitrite [163]. Administration of melatonin shortly after TBI decreases brain edema, neuronal death, and memory deficits [164, 165]. These changes are correlated with improvements in markers of oxidative damage (lipid peroxidation) and in the levels of low molecular weight antioxidants, such as vitamin C [166–169]. However, improvement is not always observed with the use of melatonin [170].

### 7.2. Carbonyl Scavengers

Besides the lipid peroxidation end products aldehydes, like MDA and 4-HNE, cells also produce continually a series of *α*-dicarbonyl species. For instance, methylglyoxal is continuously produced during glycolysis but can also be produced nonenzymatically from carbohydrates and glycation of proteins, besides other endogenous sources [171]. The production of methylglyoxal, the main dicarbonyl byproduct of glycolysis, is responsible for significant portion of protein glycation and, consequently, the induction of cellular toxicity [66]. Methylglyoxal modifies proteins to form advanced glycation end product (AGE) residues. These modified proteins can activate receptors of AGE (RAGE) leading to the upregulation and the expression of proinflammatory mediators [172, 173]. This proinflammatory cascade involving RAGE has been recently shown to be relevant to TBI. RAGE is upregulated in human brain as short as 30 min after TBI and maintained elevated up to 6 days, presenting maximal values at 24 h after TBI [174]. Importantly, the inflammatory cytokine high-mobility group box 1 (HMGB1), a RAGE ligand, was increased in CSF and could be associated with poor outcome after TBI in infants and children [175]. Since HMGB1 is released in the CSF, an anti-HMGB1 antibody strategy has proven to be effective in inhibiting fluid percussion-induced brain damage in mice [176].

This successful strategy brings attention to the potential importance of AGE on TBI. To prevent AGE production and consequent RAGE activation, *α*-dicarbonyls, like methylglyoxal, need to be eliminated or sequestered in order to avoid a negative impact in TBI outcome. Carbonyl scavengers have been used with the aim of reducing the “aldehyde load” [177] and have been investigated *in vivo* and *in vitro* regarding their effects on neuroprotection, but there is limited literature with regard to TBI [178, 179]. The carbonyl scavenger D-penicillamine binds primarily to aldehydes in an irreversible manner which inhibits their damaging effects and has also been shown to scavenge peroxynitrite as well [180]. Acute penicillamine administration has previously been shown to improve neurological recovery in the mouse concussive head injury model [181] and to protect brain mitochondria [180]. Carnosine is also a dicarbonyl scavenger with neuroprotective properties in TBI [182]. Hydralazine, an acrolein scavenger, decreased cell membrane leakage and permeability in spinal cord injury [183]. Aminoguanidine, another carbonyl scavenger and an inhibitor of inducible nitric oxide synthase (iNOS), was able to reduce carbonyl stress in diabetes, preserving neurological scores [184, 185], preventing the decrease in cortical necrotic neuron counts [186], and reducing infarct volume [185] in animal models of TBI. Aminoguanidine treatment is also able to reduce infarct size and preserve BBB in a middle cerebral artery occlusion model of ischemia [187] and reduce damage area in traumatic spinal cord injury [188], effects that are relevant to TBI.

### 7.3. Glutathione-Promoting Drugs

Strategies that aim at increasing the GSH levels have also been tested in animal models. GSH-promoting agents, like N-acetylcysteine (NAC) and *γ*-glutamylcysteine ethyl ester, are effective neuroprotectants in preclinical studies. Administration of *γ*-glutamylcysteine ethyl ester after TBI in mice preserves GSH, decreases autophagy, and improves both behavioral and histological outcomes [189]. *γ*-glutamylcysteine ethyl ester decreased levels of protein carbonyls and 3-nitrotyrosine after sever TBI [117] as well as, preserved glutathione status, endotelial function, and BBB [190].

Studies with NAC showed efficacy of this drug in decreasing the TBI-mediated oxidative stress and limiting the volume of injury in rats [191]. NAC also restored respiratory function and calcium transport when administrated 1 h after trauma [192]. In contrast, this protective effect was not present when NAC was administrated 2 h after the lesion, indicating that it must act in the early stages of the lesion. NAC was able to restore brain GSH levels from 1 h to 14 days after TBI, suggesting that this protective effect may be related to the maintenance of the GSH levels. Other studies also showed anti-inflammatory properties of NAC. NAC administration decreased several inflammatory cytokines and the activation of NF-*κ*B, which reduced brain edema, BBB permeability, and apoptotic index in the injured brain [193].

NAC was also tested in a randomized double blind, placebo-controlled study with soldiers who were exposed to ordnance blast and met the criteria for mild TBI. Patients received placebo or NAC for seven days, and the treatment showed improvement in symptoms such as dizziness, memory loss, and sleep disturbances [194]. Given the fact that NAC is already approved by the FDA, this is a good candidate for future clinical trials.

### 7.4. Steroids

Corticosteroids have been shown to inhibit the phospholipase A2, cyclooxygenase, and lipoxygenase pathways, limiting the release of arachidonic acid and its metabolites, downregulating proinflammatory cytokines, and dampening the inflammatory response [195]. Methylprednisolone is able to attenuate cellular damage by a direct antioxidant effect, thereby inhibiting lipid peroxidation and protecting cellular membranes [195]. Preclinical data showed that this steroid affects the outcome. Methylprednisolone was able to improve brain edema in rats, and this was dependent on dosing, time of administration, and method of treatment [196].

The indiscriminate use of corticosteroids was long performed without an evaluation of their devastating effects, as evidenced by clinical trials. However, The Brain Trauma Foundation guidelines published in 2000 state that “the use of corticosteroids is not recommended for improving outcome or reducing intracranial pressure in patients with severe brain injury” and “the major of available evidence indicates that steroids do nor improve outcome or lower ICP in severely head injured patients and the routine use of steroids for these purposes is not recommended” [197]. In 2004 the corticosteroid randomization after significant head injury (CRASH) study demonstrated greater mortality in patients receiving high doses of methylprednisolone compared to placebo [198]. A Cochrane meta-analysis correlated the use of corticosteroids with increased risk of gastrointestinal bleeding, hyperglycemia, and also higher mortality rates [199].

Preclinical studies, however, suggested a possible antioxidant and neuroprotective effect of physiological doses of sex hormones following TBI [200, 201]. Progesterone has been shown to decrease cerebral edema in a pluripotent manner, reducing lipid peroxidation, aquaporin expression, proinflammatory cytokine release, and complement activation [202]. Unfortunately, studies in humans have not demonstrated a significant association between gender and prognosis [203, 204]. Nevertheless, clinical trials have demonstrated better outcomes in patients treated with progesterone [205, 206]. In a phase II study, progesterone showed significant reduction in mortality and improvement in the Glasgow outcome scale at 3 and 6 months after TBI; however, results from a phase III multicentre, randomized clinical trial are pending [207]. A Cochrane systematic review concluded that “Progesterone may improve neurologic outcome of patients suffering TBI.” “…evidence is insufficient and there is a need for further studies to support the use of progesterone in the management of TBI. Further large and multicentre clinical trials on progesterone are required to assess the effect of progesterone in people with acute TBI” [206].

Another strategy to prevent ROS-mediated damage is to block the propagation of lipid peroxidation. Lazaroids (21-aminosteroids) are a class of compounds with the membrane-stabilizing properties, similar to the effect of glucocorticoids. Tirilazad inhibits lipid peroxidation through its membrane-stabilizing properties, which prevent the propagation of the reaction between one oxidized fatty acid to the next, and through free radical scavenging.

In an animal model of TBI, administration of the lazaroid tirilazad improved neurological score and survival rate when administrated shortly after the trauma (5–60 min) [208]. In a weight drop TBI model in rats, tirilazad also diminished neuron loss at 24 h and increased neuronal survival 14 days after the injury [209]. Furthermore, administration of tirilazad reduced extracellular O_2_^•−^ after TBI, which may contribute to its neuroprotective effect [210]. The protective effect of tirilazad has also been demonstrated in animal models of ischemia and subarachnoid haemorrhage, both events linked with the secondary injury pathways in TBI [211].

Tirilazad attenuated cerebral edema after TBI [212] and protected against arachidonic acid-induced vasogenic brain edema in rats by inhibiting lipid peroxidation [213]. Since tirilazad does not penetrate the BBB effectively, it is found mainly in the membranes of endothelial cells, where it could exert its action. However, it is possible that tirilazad can reach neuronal cells during the phase of increased permeability of the BBB in TBI, which can allow tirilazad to penetrate brain areas where it can act as a neuroprotectant [214].

In the 90s, tirilazad entered a phase III multicenter trial. The study comprised 1120 head-injured patients with moderate or severe TBI. Patients received tirilazad or placebo within 4 h after injury at a dose of 2.5 mg/kg every 6 h for 5 days. The trial failed to show a beneficial effect of the treatment in either moderate or severe injured groups. However, *post hoc* analysis showed that male patients that also had subarachnoid hemorrhage had significantly less mortality [215]. Meta-analysis studies questioned the efficacy of tirilazad in subarachnoid hemorrhage [216, 217]. In other clinical studies, tirilazad significantly contributed to recovery after spinal cord injury [218], but recent studies are not available. The poor penetration through the BBB might be responsible for the negative result, as well as the nature of the head injury.

### 7.5. Calcium Blockers and Immunosuppressants

Calcium blockers and immunosuppressants that could limit the production of ROS/RNS by membrane stabilization or reduction of inflammation have also been investigated. Calcium-channel antagonists, such as nimodipine, would block the effects of the influx of calcium into the cell after TBI. Unfortunately, in an extensive Cochrane meta-analysis, nimodipine only reduced the risk of death in a subgroup of patients with subarachnoid hemorrhage [219]. Cyclosporine A, a known immunosuppressant, is thought to mediate neuroprotection by decreasing the mitochondrial permeability and therefore the influx of Ca^2+^, organelle swelling, and cell death [34]. Cyclosporine A also showed promising results in a phase II study; however, validation by a multicentre, randomized phase III clinical trial is pending [207].

### 7.6. Other Drugs

Interference of other pathways which result in the production of ROS/RNS has thus far failed to produce good clinical outcomes. Bradykinin antagonists such as Deltibant [220], modulators of excitotoxicity, and glutamate such as dexanabinol [221, 222], magnesium sulfate [223], and selfotel [224] have all been proven to be ineffective in clinical trials [207].

### 7.7. Nrf2/ARE: A Putative Therapeutic and Biomarker Route

Activation of the antioxidant response element (ARE) has been implicated in neuroprotection, as this induces expression of genes involved in decreasing oxidative stress, inflammatory damage, and accumulation of toxic metabolites [225]. Several transcriptional factors can bind to ARE, including the nuclear factor-erythroid 2-related (Nrf2) protein that activates transcription in response to oxidative stress or electrophilic attack [226]. Nrf2 is a basic leucine zipper redox-sensitive transcription factor reported to be a pleiotropic regulator of cell survival mechanisms [227]. Recent studies have demonstrated that Nrf2 plays an indispensable role in the upregulation of Nrf2-dependent antioxidant enzymes and the reduction of oxidative damage after TBI [228]. Under basal conditions, Nrf2 is sequestered in the cytoplasm by the cytosolic regulatory protein Keap1. In conditions of oxidative or xenobiotic stress, Nrf2 translocates to the nucleus where it binds to ARE, neutralizing the BACH1 competitive inhibition, activating this promoter, and resulting in transcription of a number of antioxidant proteins [229, 230].

Several antioxidants from the diet which have been studied for their neuroprotective properties are proposed to function via the activation of Nrf2/ARE. Caffeic acid phenethyl ester is an active component of bee propolis extracts which displays anti-inflammatory, immunomodulatory, antiproliferative, and antioxidant properties. Caffeic acid phenethyl ester treatment decreased MDA levels and increased GPx and SOD activity in a rat experimental model of TBI [231]. Green tea is rich in polyphenols that have important antioxidant activity. Epigallocatechin gallate treatment in rat models of TBI decreased the free radical burden (e.g., O_2_^•−^ and ^•^OH) induced by brain injury. This antioxidant effect decreased tissue damage induced by free radicals, including a decrease of neuronal cell degeneration, apoptotic cell death around the damaged area, and improved brain function (water maze) [232–234]. Ginseng, from the root of *Panax ginseng*, is a well-known traditional herbal medicine that has been used widely for thousands of years. The ginseng saponins are generally considered as the principal bioactive ingredients. Preclinical studies suggested that the neuroprotective effects of ginseng saponins are potentially associated with protection against oxidative stress damage [235–238]. The flavonoid quercetin improved neuronal electrical activity and decreased proinflammatory effects in a model of TBI [239]. Resveratrol is a polyphenolic compound enriched in grapes and red wine. Resveratrol has been shown to be a promising neuroprotective agent in TBI models, possibly inhibiting lipid peroxidation [240–242]. The use of resveratrol in an experimental model of TBI was able to counteract oxidative damage and prevent the depletion of the antioxidant glutathione and also resulted in a reduction of infarct area [110]. Polyphenolic derivatives of curcumin have also been shown to protect the brain against the effects of experimental TBI by decreasing oxidative stress [44, 45, 243, 244]; however, the observed effects may be attributable not only to the antioxidant properties of flavonoids but also to a response occurring secondary to Nrf2/ARE activation. Sulforaphane, similar to other flavonoids, is an Nrf2/ARE signaling activator and is present in cruciferous vegetables such as broccoli. Nrf2/ARE activation by sulforaphane treatment after experimental TBI was confirmed by induction of target genes such as glutathione S-transferase *α*3 and heme oxigenase-1 and associated with an improvement in BBB integrity [245]. The administration of sulforaphane is also neuroprotective in various animal models of TBI, specifically reducing cerebral edema and oxidative stress and thus decreasing cognitive deficits [246, 247]. In the spinal cord model, sulforaphane was able to produce both rapid (30 min) and long-lasting (3 days) responses, also corroborated by the induction of Nrf2/ARE target proteins, including the rate-limiting enzyme for glutathione synthesis (GCL), heme oxygenase 1, and NADPH quinonereductase-1, which cooperate to decrease levels of the proinflammatory markers TNF-*α* and IL-1*β* [248].

There is some evidence to support the idea that Nrf2 activation is able to protect against dicarbonyl stress by induction of glyoxalase 1 [249], the enzyme catalyzing the reaction of methylglyoxal with glutathione. It has been suggested that methylglyoxal and glyoxalase 1 can also modulate seizure intensity as metabolic sensors [250], which may have implications in TBI, since hyperglycolysis (high flux through anaerobic glycolysis) after TBI is associated with a negative outcome [251–253]. Hyperglycolysis may lead to increased production of methylglyoxal [254, 255]. Nrf2 activation would alleviate dicarbonyl stress by inducing glyoxalase 1 and glutathione synthesis [249]. In fact, resveratrol was effective in protecting hepatic (Hep G2) cells against methylglyoxal toxicity due induction of Nrf2, which leads to increased expression of glyoxalase I and antioxidant defenses [256]. The flavones Fisetin was highly protective against carbonyl stress in the Akita mice, a model of diabetes type 1, by inducing Nrf2-dependent enzymes such as glyoxalase and glutamate-cysteine ligase, the rate limiting enzyme in glutathione synthesis [257]. Fisetin was also neuroprotective in an ischemia model [258]. Quercetin displays a strong antiglycation action when albumin was incubated with methylglyoxal. Curcumin, despite being a potent Nrf2 inducer and effective as a neuroprotectant in TBI, is a strong inhibitor (IC(50) ~10 *μ*M) of glyoxalase I [259], which would jeopardize its possible antiglycation properties. Nevertheless, curcumin is as potent dicarbonyl scavenger [260]. Caffeic acid was effective in protecting proteins against AGE formation *in vitro* [261] and epigallocatechin-3-gallate has been proposed as a dicarbonyl scavenger [262], as well as other phenolic antioxidants [263]. In this regard, flavonoids are added to the list of carbonyl scavengers, acting directly as scavenger or indirectly through activation Nrf2-dependent antiglycation enzymes such as glyoxalase I.

The use of antioxidants which sequester ROS and other harmful molecules is a promising strategy to increase neuroprotection in TBI [211, 240]; however, part of the antioxidant effect of such exogenous substances like flavonoids may be due to the induction of endogenous antioxidants. Nrf2 activators may be prime candidates for the attenuation of oxidative stress and subsequent neurotoxicity induced by TBI.

## 8. Concluding Remarks

Improving TBI outcomes will greatly reduce the heavy societal and economic burden currently associated with this condition. Oxidative stress appears to be a primary driver of TBI pathophysiology, and while several stress-related markers and new therapeutic drugs with distinct antioxidative stress mechanisms have been proposed for the diagnosis and treatment of TBI, clinical consolidation based on proven efficacy is still necessary. Research prospects for new biomarkers of TBI should focus on demonstration of functionality in converging pathways that can impact multiple pathophysiological cascades.

As part of a consistent and clinically applicable intervention, we believe it would be crucial to not only limit the development of secondary damage following TBI but also to activate major neuroprotective pathways. The identification of relevant biochemical markers along with successful therapeutic targeting in experimental models of TBI has demonstrated the importance of several regulatory pathways in neuroprotection, in particular Nrf2/ARE. This pathway is activated by oxidative stress and nitric oxide production, which are also both points of convergence in TBI-related pathophysiological cascades.

Regarding the inhibition of secondary damage, the toxic effect of aldehydes can be mitigated by aldehyde scavengers, but also by upregulation of endogenous detoxification and antioxidant defenses, which can limit damage to DNA, lipids, and proteins. However, other pathophysiological routes, like inflammation, also need to be targeted to reduce damage.

Our opinion is that the ideal, clinically feasible TBI therapy would involve the targeting of a pathway which converges on multiple pathophysiologic processes in TBI, such as Nrf2/ARE, and which can also be modulated by external factors like dietary substances. Such a pleiotropic drug would have the potential to usher in a new era of effective neuroprotection for traumatic brain injury.
